# Impact of protein-energy malnutrition on outcomes of patients with sickle cell disease: an analysis of the National inpatient sample

**DOI:** 10.1007/s00277-025-06358-2

**Published:** 2025-04-21

**Authors:** Mrinalini Ramesh, Yasmin Fakhari-Tehrani, Vaishali Deenadayalan, Parikshit Padhi

**Affiliations:** 1 Department of Internal Medicine, University at Buffalo, Buffalo, NY 14203 USA; 2 Department of Hematology-Oncology, Roswell Park Cancer Institute, Buffalo, NY 14203 USA; 3 Department of Internal Medicine, (Division of Hematology and Oncology), Jacobs School of Medicine and Biomedical Sciences, University at Buffalo, Buffalo, NY 14203 USA

**Keywords:** Adverse inpatient outcomes, Disparities, Sickle cell disease, Hematology

## Abstract

Patients with sickle cell disease (SCD) are often malnourished, leading to complications such as increased vaso-occlusive crises, infections, prolonged hospital stays, and decreased quality of life. This study examines the impact of protein-energy malnutrition (PEM) on clinical outcomes in hospitalized SCD patients. A retrospective cohort study using National Inpatient Sample data (2016–2020) was performed. Adult SCD patients were stratified by PEM status. Primary outcomes included mortality, length of stay, and hospital charges. Multivariate regression analyses were performed in STATA 17. Among 771,175 SCD patients, 25.9% (20,030) had PEM. More PEM patients were female (57.3%), and PEM was more prevalent in males compared to those without (42.7% vs. 34.3%, *p* < 0.001). Patients with PEM had higher Charlson Comorbidity Index scores (≥ 3: 42.6% vs. 12.8%, *p* < 0.001). PEM was associated with increased mortality (aOR 2.66, *p* < 0.001), longer hospital stays (9.56 vs. 4.79 days, *p* < 0.001), and higher costs ($100,209 vs. $41,412, *p* < 0.001). PEM also raised the odds of intubation, pressor support, acute kidney injury, sepsis, blood transfusion, pneumonia, and urinary tract infections (all *p* < 0.001). PEM is associated with worse outcomes in hospitalized SCD patients, leading to higher mortality, extended stays, and increased costs due to intensive interventions. Early nutritional consultation may improve patient outcomes.

**Supplementary Information:**

The online version contains supplementary material available at 10.1007/s00277-025-06358-2.

## Introduction

Sickle Cell Disease is a hereditary condition stemming from a genetic mutation in the β-globin gene, resulting in the generation of aberrant hemoglobin S. This disorder impacts the morphology and functionality of erythrocyte, transforming them into a sickle or crescent-like shape [1]. This can lead to a range of acute and chronic complications, including hemolysis and anemia, vaso-occlusive crises, acute chest syndrome, increased risk of thromboembolism, and organ infarction. Furthermore, patients are at a heightened risk of severe infections. These complications result in elevated morbidity and mortality within this patient population, as well as an increased likelihood of hospitalizations [2]. Patients with sickle cell disease experience markedly increased energy and protein requirements due to the persistent inflammatory state, heightened metabolic activity, and substantial energy expenditure associated with the continuous turnover and rapid destruction of their red blood cells. This amplified nutritional need is crucial for supporting the body’s attempts to maintain homeostasis and address the numerous complications of this complex, debilitating hematological disorder [3]. Protein-energy malnutrition (PEM) is a frequent condition observed in patients with Sickle Cell Disease, marked by diminished muscle mass, impaired growth, and suboptimal weight progression [4]. Complications associated with sickle cell disease, including recurrent infections, persistent inflammation, and oxidative stress, lead to heightened nutritional requirements and elevated protein breakdown. Protein-energy malnutrition impairs the synthesis of hemoglobin and essential enzymes responsible for managing oxidative stress, further exacerbating anemia and oxidative damage in sickle cell disease. This creates a detrimental feedback loop, where malnutrition exacerbates the severity of sickle cell disease, while the complications of sickle cell disease continue to worsen nutritional status [5, 6].

In this study we examine the impact of protein-energy malnutrition on the overall clinical outcomes of hospitalized patients with sickle cell disease, including their length of hospital stay, complication rates, mortality, and other relevant health indicators. This information will help guide clinicians in recognizing and managing this critical condition and provide a basis for future research to address the malnutrition complications associated with sickle cell disease. This may allow further guidelines to bridge the gap between nutritional science and clinical practice, ultimately improving the quality of life for SCD patients.

## Materials & methods

This study used publicly available, deidentified data and was deemed exempt from Institutional Review Board (IRB) approval.

### Study design and data source

This retrospective, cross-sectional study utilized data from the National Inpatient Sample (NIS), the largest all-payer inpatient care database in the United States, maintained by the Healthcare Cost and Utilization Project (HCUP) [7]. The NIS contains discharge data from approximately 7 million hospital stays each year, representing a 20% stratified sample of community hospitals nationwide. This database offers researchers a valuable resource to analyze the patterns of healthcare delivery and outcomes within a nationally representative sample of hospitalized patients in the US. Hospitals were stratified based on ownership, urban or rural area, geographic region, and bed size. All discharges from these hospitals were weighted to be nationally representative and cover more than 97% of the US population. The dataset was de-identified and contained information on patient demographics, diagnoses, procedures, hospital characteristics, and outcomes. The study period covered January 1, 2016, to December 31, 2020. The International Classification of Diseases, Tenth Revision, Clinical Modification/Procedure Coding System (ICD-10-CM/PCS) was used for coding diagnoses and procedures in the NIS data. The specific ICD codes were derived from the papers by Deenadayalan et al. [8, 9]. Diagnoses were categorized as principal or secondary. The principal diagnosis was the main ICD-10 code responsible for the hospitalization, while secondary diagnoses included any additional ICD-10 codes relevant to the patient’s condition but not the primary reason for admission. It is important to note that NIS data do not provide a reliable mechanism to distinguish secondary diagnoses coded during the index hospital admission from those coded in previous admissions.

### Study population and inclusion criteria

We included adult patients (aged 18 years and older) admitted to acute care hospitals in the United States during the study period who had a principal diagnosis of sickle cell disease, as identified by ICD-10-CM/PCS. To create our primary comparison groups, we utilized PEM as a secondary diagnosis within the initial population of SCD patients. This resulted in two distinct groups: patients admitted with a primary diagnosis of SCD with a secondary diagnosis of PEM, and patients admitted with a primary diagnosis of SCD without PEM. The NIS cannot distinguish between individual patients. Consequently, the population under investigation was not composed of distinct patients but rather instances of admissions for SCD. As a result, multiple admissions could potentially involve the same patient.

### Variables and outcomes

The primary independent variable was the presence of PEM. Other independent variables included patient demographics (age, sex, race/ethnicity), insurance type, and hospital characteristics (size, location, region). In our analysis, we examined various patient attributes, including demographic information, characteristics of the hospital, and pertinent medical comorbidities. The primary outcome was all-cause in-hospital mortality. The secondary outcomes were the effects of our primary comparison group (presence of PEM) on the length of stay and the hospital charges of hospitalized patients for SCD diagnosis. Outcomes were defined according to the International Classification of Diseases, Tenth Revision, Clinical Modification (ICD-10-CM) codes, and HCUP’s Clinical Classifications Software for diagnoses and procedures. Supplementary Table 1 (Table S1) contains a list of the utilized ICD-10 codes that are pertinent to the study.

### Statistical analysis

Data were analyzed using STATA software version 17 (StataCorp, College Station, TX). We conducted individual univariate logistic regression analyses using all available variables to obtain crude data. Univariate analysis was performed using variables including age, sex, race, Charlson Comorbidity Index, insurance type, ZIP code, hospital region, bed size, and hospital location. Crude odds ratios were obtained from these analyses. Variables with a P-value < 0.1 were included in a multivariate logistic regression model to account for potential confounding factors. For in-hospital mortality and secondary outcomes, including length of stay and hospital charges, UTI, sepsis, intubation, neutropenia, AKI, ARF, pneumonia and blood transfusion, adjusted odds ratios were derived using a multivariate logistic regression model. Adjusted odds ratios were used to account for confounders and provide a more accurate estimate of the independent effect of the primary exposure on the outcome. Specifically, we controlled for age and gender due to their association with hospital outcomes in sickle cell patients [10]. The Charlson Comorbidity Index was included to adjust for baseline comorbid conditions, which may impact both treatment decisions and patient outcomes [11]. Hospital bed size and region were incorporated to account for possible differences in healthcare facility capacity and geographic variability in care delivery [12]. Race and insurance type were included to adjust for potential disparities in healthcare access and utilization [10, 12]. Income was considered to reflect socioeconomic status, which can influence both healthcare-seeking behavior and treatment options [8]. Continuous variables were compared using the independent sample t test, while categorical variables were analyzed using Pearson’s chi-square and Fisher’s exact tests. All tests were two-sided. Statistical significance was determined for outcomes with P values < 0.05 and a 95% confidence interval.

## Results

A total of 771,175 adults with SCD were identified during the study period from 2016 to 2020. Of these patients, 25.9% (20,030) had PEM. Demographic characteristics, including mean age, sex, and race, are summarized in Table 1. As expected, the largest racial group in the study population included Black patients. A higher proportion of patients diagnosed with PEM were female (57.3%) compared to males (42.7%). However, there were more male patients with a diagnosis of PEM than without (42.7% vs. 34.3%, *p* < 0.001). The average income between the two cohort groups was similar, indicating no socioeconomic differences. Regarding insurance, SCD patients without PEM were more likely to be on Medicaid and have private insurance, while Medicare was the most prevalent insurance type among those patients with PEM. Analysis of the Charlson Comorbidity Index, which predicts 10-year survival in patients with multiple comorbidities, was associated with higher scores in patients with PEM than without (42.6% vs. 12.8%, *P* < 0.001), as noted in Table 1.

**Table 1:** Demographics, comorbidities and hospital admission data among SCD patients with and without a diagnosis of PEM

	SCD without PEM (*n* = 751,145)	SCD with PEM (*n* = 20,030)	*P* value
**Age** > 18 - <45 >=45 to < 65 >=65	584,391 (77.8%) 130,699 (17.4%) 36,055 (4.8%)	9,855 (49.2%) 6,590 (32.9%) 3,585 (17.9%)	< 0.001
**Mean age**	35.5	46.4	< 0.001
**Sex** Female Male	493, 502 (65.7%) 257,643 (34.3%)	11,477 (57.3%) 8553 (42.7%)	< 0.001
**Charlson Comorbidity Index**			< 0.001
0	422,144 (56.2%)	4927 (24.6%)	
1	169,759 (22.6%)	3585 (17.9%)	
2	63,847 (8.5%)	2985 (14.9%)	
>/=3	96,147 (12.8%)	8533 (42.6%)	
**Comorbidities**			
Diabetes Mellitus	72,861 (9.7%)	3846 (19.2%)	< 0.001
Hypertension	117,179 (15.6%)	4046 (20.2%)	< 0.001
Smoking	201,307 (26.8%)	7011 (35.0%)	< 0.001
Congestive Heart Failure	59,340 (7.9%)	3826 (19.1%)	< 0.001
Chronic Kidney Disease	80,373 (10.7%)	5729 (28.6%)	< 0.001
Obesity	100,653 (13.4%)	1582 (7.9%)	< 0.001
Dyslipidemia	56,336 (7.5%)	2864 (14.3%)	< 0.001
Coronary Artery Disease	36,055 (4.8%)	1763 (8.8%)	< 0.001
Cerebrovascular accident	16,525 (2.2%)	1042 (5.2%)	< 0.001
COPD	31,548 (4.2%)	2023 (10.1%)	< 0.001
**Race**			< 0.001
White	15,774 (2.1%)	621 (3.1%)	
Black	692,556 (92.2%)	18,448 (92.1%)	
Hispanic	37,557 (5.0%)	821 (4.1%)	
Other	3756 (0.5%)	120 (0.6%)	
**Insurance**			< 0.001
1-Medicare	210,321 (28.0%)	8994 (44.9%)	
2-Medicaid	344,776 (45.9%)	7131 (35.6%)	
3-Private	164,501 (21.9%)	3285 (16.4%)	
4-Self pay	30,797 (4.1%)	601 (3.0%)	
**Hospital Region**			< 0.001
Northeast	153,985 (20.5%)	3966 (19.8%)	
Midwest	142,718 (19.0%)	4727 (23.6%)	
South	391,347 (52.1%)	8673 (43.3%)	
West	62,345 (8.3%)	2664 (13.3%)	
**Hospital Bedsize**			< 0.001
Small	127,695 (17.0%)	3385 (16.9%)	
Medium	200,556 (26.7%)	4407 (22.0%)	
Large	422,144 (56.2%)	12,218 (61.0%)	
**Hospital Location**			< 0.001
Rural	26,290 (3.5%)	461 (2.3%)	
Urban	724,855 (96.5%)	19,569 (97.7%)	
**Income status** (based on Median household income quartiles for patient’s ZIP Code)			0.071
$1 - $45,999	376,324 (50.1%)	9895 (49.4%)	
$46,000 - $58,999	169,759 (22.6%)	4447 (22.2%)	
$59,000 - $78,999	125,441 (16.7%)	3305 (16.5%)	
$79,000 or more	78,870 (10.5%)	2404 (12.0%)	

**Table 2:** Complications associated with patients with SCD during their admission

	SCD without PEM (*n* = 751,145)	SCD with PEM (*n* = 20,030)	*P* value
**Complications**			
Urinary Tract Infection	27,792 (3.7%)	1975 (9.9%)	< 0.001
Sepsis	18,028 (2.4%)	2223 (11.1%)	< 0.001
Intubation	8263 (1.1%)	1202 (6.0%)	< 0.001
Pressor requirement	2253 (0.3%)	321 (1.6%)	< 0.001
Acute Kidney Injury	58,589 (7.8%)	4747 (23.7%)	< 0.001
Acute Respiratory Failure	24,037 (3.2%)	1943 (9.7%)	< 0.001
Neutropenia	18,779 (2.5%)	601 (3.0%)	0.02
Pneumonia	54,082 (7.2%)	2864 (14.3%)	< 0.001
Blood Transfusion	126,192 (16.8%)	5268 (26.3%)	< 0.001

A total of 0.67% (*N* = 5130) patients with SCD died, among which 740 (0.58%) had PEM. Further analyses indicated that PEM was associated with twofold increased odds of mortality (adjusted odds ratio [aOR] 2.66, *p* < 0.001, 95% confidence interval [CI] 2.17–3.26). Additionally, patients with PEM were associated with a longer length of stay (9.56 vs. 4.79 days, adjusted difference of 4.08 days, *p* < 0.001, 95% CI 3.74–4.42) and higher total charges ($100,209 vs. $41,412, adjusted difference of $46,004, *p* < 0.001, 95% CI $39,879–$52,128).

The presence of PEM was also associated with increased odds of several secondary outcomes, including intubation (aOR 2.98, *p* < 0.001, 95% CI 2.58–3.49), pressor support (aOR 2.94, *p* < 0.001, 95% CI 2.16–4.00), acute kidney injury (AKI) (aOR 1.64, *p* < 0.001, 95% CI 1.49–1.81), sepsis (aOR 3.26, *p* < 0.001, 95% CI 2.90–3.66), blood transfusion (aOR 1.58, *p* < 0.001, 95% CI 1.45–1.72), neutropenia (aOR 1.53, *p* < 0.001, 95% CI 1.26–1.86), pneumonia (aOR 1.85, *p* < 0.001, 95% CI 1.68–2.05), and urinary tract infection (UTI) (aOR 2.30, *p* < 0.001, 95% CI 2.04–2.59) compared to the other cohort, as noted in Table 2.

## Discussion

This study evaluated hospital outcomes for adults with sickle cell disease (SCD) and protein-energy malnutrition (PEM), a prevalent comorbidity that remains underexplored in the literature. Our analysis found that 25.9% of adults with SCD had concurrent PEM, which is a significant proportion of this vulnerable patient group. Our study population was predominantly Black (Table 1), aligning with the demographic characteristics typically seen in SCD populations [13].

A particularly concerning result from our study was the higher burden of comorbidities among patients with both SCD and PEM. Specifically, these patients were associated with higher Charlson Comorbidity Index (CCI) scores, reflecting an increased burden of chronic disease. This elevated CCI suggests that patients with SCD and PEM may be more susceptible to complications, a hypothesis supported by the observed increase in secondary adverse outcomes in this cohort. These findings are consistent with broader evidence indicating that malnutrition is associated with adverse clinical outcomes across various patient populations [14]. This underscores the critical need for early detection and management of PEM in individuals with SCD to prevent the exacerbation of other health conditions.

Our study also demonstrated an associated increase in the mortality risk in patients with PEM. The odds of mortality were over twofold higher for patients with SCD and PEM (aOR 2.66, *p* < 0.001). This finding aligns with existing evidence suggesting that malnutrition exacerbates the severity of SCD complications including the frequency and severity of vaso-occlusive crises and impairs recovery from acute crises [3]. Nutritional deficiencies particularly in key nutrients such as zinc, vitamin D, and B vitamins are associated with increased disease severity and complications [15]. Zinc, in particular, plays a crucial role in immune function and cellular repair, contributing to more frequent and severe vaso-occlusive crises [15]. Malnutrition can also impair immune function, making patients more susceptible to infections, which are a significant cause of morbidity and mortality in SCD [16]. These findings highlight the urgency of addressing PEM in this population, as malnutrition may directly contribute to poor outcomes such as increased infection risk, prolonged hospital stays, and a higher risk of death.

Patients with SCD and PEM were also associated with longer hospitalizations (an adjusted increase of 4.08 days) and higher total charges compared to those without PEM. The extended hospital stays, and elevated healthcare costs underscore the impact of PEM on patient recovery, suggesting that addressing nutritional deficiencies could potentially reduce hospital resource utilization. These results align with studies in other populations, which have consistently shown that malnutrition increases both the length of hospital stay and the cost of care [17]. Ruiz et al. demonstrated that malnutrition in hospitalized patients is associated with increased length of stay (LOS) and higher hospital costs [18]. Similarly, Curtis et al. found that malnourished patients had longer hospital stays and incurred higher costs compared to well-nourished patients [19]. Studies in children with sickle cell disease have also shown that inadequate nutritional intake and lower body mass index are significantly associated with an increased number of hospitalizations and longer hospital stays [20]. It is imperative that hospital systems recognize the financial and clinical implications of PEM and implement appropriate nutritional interventions to mitigate these effects.

Furthermore, our study identified several complications that had higher odds of occurring in patients with SCD and PEM, including intubation, vasopressor support, acute kidney injury (AKI), sepsis, blood transfusions, neutropenia, pneumonia, and urinary tract infections (UTIs). These findings suggest that malnutrition is associated with an increased risk of systemic failure and poor recovery in hospitalized patients, making them more vulnerable to infections and organ dysfunction. A review of the impact of malnutrition on patients with SCD found that nutritional deficiencies in this population cause significant complications including pain crises, infections, and decreased quality of life [3]. Importantly, these secondary outcomes also highlight the potential for PEM to act as a modifiable risk factor in patients with SCD, where improving nutritional status could help reduce the incidence of these complications.

The pathophysiological mechanisms linking PEM with these outcomes in SCD are multifactorial. Malnutrition, particularly deficiencies in key nutrients like protein and micronutrients, can impair immune function, reduce tissue repair capacity, and compromise vascular health, all of which are critical in the context of SCD [16]. The interplay between these factors likely contributes to the increased risk of complications observed in this study.

There are several limitations to our study. First, the observational design of the study design prevents us from establishing causality. While our findings indicate strong associations between PEM and poor outcomes, the study cannot definitively determine whether nutritional interventions would improve these outcomes. It is also important to note that patients can have repeated admissions recorded as separate hospital admissions, which can lead to overrepresentation of certain individual patients. Furthermore, the data utilized were derived from administrative claims, which may lack granularity regarding the severity of PEM and SCD. Regarding this, we are unable to determine the exact nutritional deficiency that could contribute to this clinical picture, including the inability to measure exact levels of albumin, micronutrient levels, vitamins or zinc. The retrospective design and reliance on administrative data via ICD-10 coding can introduce potential misclassification biases. The PEM diagnosis may not be consistently captured across hospitals, and disease severity is not directly measurable. The lack of longitudinal follow-up also prevents assessment of long-term outcomes related to PEM in SCD patients. Also, while we controlled a variety of demographic and clinical factors, residual confounding remains a possibility including an individual patient’s severity of anemia, history of prior hospitalizations and the impact of social determinants of health.

## Conclusion

In conclusion, our study highlights the impact of PEM on hospital outcomes in adults with SCD. The association with increased mortality, prolonged hospital stays, higher healthcare costs, and a greater likelihood of severe complications underscores the need for integrated care approaches that address both SCD and malnutrition. Future research should focus on exploring targeted interventions to prevent or treat PEM in this high-risk population to potentially reduce the burden of disease and improve clinical outcomes.

## Electronic supplementary material

Below is the link to the electronic supplementary material.

Supplementary Material 1

## Data Availability

All the data used for this study is from the publicly available National Inpatient Sample (NIS) database.
