# Supplementary material for: Impact of protein-energy malnutrition on outcomes of patients with sickle cell disease: an analysis of the National inpatient sample
Source: Ann Hematol. 2025 Apr 21;104(4):2171–7. doi: 10.1007/s00277-025-06358-2 (PMC12053322; doi:10.1007/s00277-025-06358-2)
Supplement: Supplementary file 1 — Supplementary Material 1 [file 277_2025_6358_MOESM1_ESM.docx]

**SUPPLEMENTARY MATERIALS**

Table S1: ICD codes for associated diagnoses used to obtain data

| **Variables** | **ICD codes** |
| --- | --- |
| Sickle cell disease | D57 |
| Intubation | 0BH13EZ, 0BH17EZ, 0BH18EZ, 0B110F4, 0B113F4, 0B114F4 |
| Mechanical Ventilation | 5A1935Z, 5A1945Z, 5A1955Z |
| Use of pressors | 3E030XZ, 3E033XZ, 3E040XZ, 3E043XZ |
| Blood transfusion | 302 |
| Anemia | D50, D51, D52, D53, D55, D56, D57, D58, D59, D60, D61, D62, D63, D64 |
| Protein Energy Malnutrition | E40, E41, E42, E43, E44, E45, E46, O25 |
| Diabetes Mellitus | E10, E11 |
| Hypertension | I10 |
| History of Tobacco smoking | Z87891, F17, Z720 |
| Congestive Heart failure | I50 |
| Chronic Kidney Disease | N18 |
| Dyslipidemia | E78 |
| Obesity | E6601, E6609, E661, E662, E668, E669, O992, Z683, Z684, Z6854 |
| Hemodialysis | Z992 |
| Coronary Artery Disease | I25 |
| Cerebrovascular Accident | I69 |
| Chronic Obstructive Pulmonary Disease | J41, J42, J43 , J44 |
| Acute Kidney Injury | N17 |
| Sepsis | A40, A41, R652, T8112 |
| Respiratory Failure | J960 |
| Neutropenia | D709, D701, D702, R5081 |
| Pneumonia | J12, J13, J14, J15, J17, J18, J16 |
| Urinary Tract Infection | N390 |
| ICD indicates International Classification of Diseases. | |
